# SIRM-SIN-AIOM: appropriateness criteria for evaluation and prevention of renal damage in the patient undergoing contrast medium examinations—consensus statements from Italian College of Radiology (SIRM), Italian College of Nephrology (SIN) and Italian Association of Medical Oncology (AIOM)

**DOI:** 10.1007/s11547-022-01483-8

**Published:** 2022-03-18

**Authors:** Antonio Orlacchio, Carlo Guastoni, Giordano Domenico Beretta, Laura Cosmai, Michele Galluzzo, Stefania Gori, Emanuele Grassedonio, Lorena Incorvaia, Carmelita Marcantoni, Giuseppe Stefano Netti, Matteo Passamonti, Camillo Porta, Giuseppe Procopio, Mimma Rizzo, Silvia Roma, Laura Romanini, Fulvio Stacul, Alice Casinelli

**Affiliations:** 1 Radiology Unit, Department of Surgical Science, University of Rome “Tor Vergata”, Rome, Italy; 2 Emergency Radiology, Policlinico Tor Vergata, Viale Oxford, 81, 00133 Rome, Italy; 3 Nephology Unit, ASST Ovest Milanese, Legnano, Italy; 4 Department of Oncology, Humanitas Gavazzeni, Bergamo, Italy; 5 Nephology Unit, Azienda Ospedaliera San Carlo Borromeo di Milano, Milan, Italy; 6 Emergency Radiology, Azienda Ospedaliera San Camillo-Forlanini, Rome, Italy; 7 Department of Oncology, IRCC Ospedale Sacro Cuore Don Calabria, Negrar Valpolicella, Italy; 8 Department of Radiology, Policlinico Giaccone University, Palermo, Italy; 9 Department of Oncology, University of Palermo, Palermo, Italy; 10 Nephology UnitSan Marco Hospital, Azienda Ospedaliero Universitaria, Catania, Italy; 11 Clinical Pathology, University of Foggia, Ospedali Riuniti, Foggia, Italy; 12 Radiology Unit, Ospedale di Oglio Po. ASST Cremona, Cremona, Italy; 13 Oncology Unit, University of Bari, Bari, Italy; 14 Genito-Urinary Medical Oncology, Fondazione Istituto Nazionale Tumori, Milano, Italy; 15 Oncology Unit, Hospital Santa Chiara, Trento, Italy; 16 Radiology Unit, Hospital F. Spaziani, Frosinone, Italy; 17 Radiology Unit, Ospedale di Cremona, ASST Cremona, Cremona, Italy; 18 Department of Radiology, Maggiore Hospital, Azienda Sanitaria Universitaria Integrata di Trieste, Trieste, Italy

**Keywords:** Consensus, Kidney injury, Nephrotoxicity, Diagnostic, Radiology, Nephrology, Oncology

## Abstract

The increasing number of examinations and interventional radiological procedures that require the administration of contrast medium (CM) in patients at risk for advanced age and/or comorbidities highlights the problem of CM-induced renal toxicity. A multidisciplinary group consisting of specialists of different disciplines—radiologists, nephrologists and oncologists, members of the respective Italian Scientific Societies—agreed to draw up this position paper, to assist clinicians increasingly facing the challenges posed by CM-related renal dysfunction in their daily clinical practice.

The major risk factor for acute renal failure following CM administration (post-CM AKI) is the preexistence of renal failure, particularly when associated with diabetes, heart failure or cancer.

In accordance with the recent guidelines ESUR, the present document reaffirms the importance of renal risk assessment through the evaluation of the renal function (eGFR) measured on serum creatinine and defines the renal risk cutoff when the eGFR is < 30 ml/min/1.73 m2 for procedures with intravenous (i.v.) or intra-arterial (i.a.) administration of CM with renal contact at the second passage (i.e., after CM dilution with the passage into the pulmonary circulation).

The cutoff of renal risk is considered an eGFR < 45 ml/min/1.73 m2 in patients undergoing i.a. administration with first-pass renal contact (CM injected directly into the renal arteries or in the arterial district upstream of the renal circulation) or in particularly unstable patients such as those admitted to the ICU.

Intravenous hydration using either saline or Na bicarbonate solution before and after CM administration represents the most effective preventive measure in patients at risk of post-CM AKI. In the case of urgency, the infusion of 1.4% sodium bicarbonate pre- and post-CM may be more appropriate than the administration of saline.

In cancer patients undergoing computed tomography, pre- and post-CM hydration should be performed when the eGFR is < 30 ml/min/1.73 m2 and it is also advisable to maintain a 5 to 7 days interval with respect to the administration of cisplatin and to wait 14 days before administering zoledronic acid.

In patients with more severe renal risk (i.e., with eGFR < 20 ml/min/1.73 m2), particularly if undergoing cardiological interventional procedures, the prevention of post-CM AKI should be implemented through an internal protocol shared between the specialists who treat the patient.

In magnetic resonance imaging (MRI) using gadolinium CM, there is a lower risk of AKI than with iodinated CM, particularly if doses < 0.1 mmol/kg body weight are used and in patients with eGFR > 30 ml/min/1.73 m2. Dialysis after MRI is indicated only in patients already undergoing chronic dialysis treatment to reduce the potential risk of systemic nephrogenic fibrosis.

## Introduction

At the present time, the number of diagnostic and interventional radiology examinations with contrast agents is increasing all over the world.

Current contrast agents are safer than those used in the recent past. However, they can cause a risk of renal impairment. Acute alterations in renal function after contrast agent administration are more common with the use of iodine-based, but they have been described occasionally with the use of gadolinium-based ones [1].

The occurrence of post-contrast acute kidney injury leads to longer hospital stay and increasing costs, morbidity and mortality [2]. The increasing rate of this complication is due to imaging examinations requiring the administration of contrast agent and in particular in patients with risk factors for contrast medium nephropathy, such as chronic renal failure, advanced age, dehydration, diabetes, severe cardiovascular disease, concomitant nephrotoxic drugs (including NSAIDs—nonsteroidal anti-inflammatory drugs, some antibiotics, some oncological drugs and cyclosporine) and repeated radiological examinations for oncological controls.

Over the years, knowledge of risk factors and implementation of preventive actions have improved, allowing to reduce the incidence of renal damage after the administration of contrast agent [3–8]. This paper will discuss risk factors of renal damage, especially related to iodine-based contrast agents, and analyze preventive actions to assess and reduce the risk of acute renal failure in clinical practice, considering that it may be due to other causes.

## Definitions and terminology

- Post-contrast acute kidney injury (PC-AKI)—deterioration of renal function within 48 h after intravascular administration of contrast agent.
- Contrast-induced acute kidney injury (CI-AKI)—the term is reserved for cases in which a causal relationship between the administered contrast agent and the deterioration of renal function can be demonstrated.

According to the KDIGO (Kidney Disease: Improving Global Outcomes) guidelines, PC-AKI is diagnosed in case of an absolute increase in serum creatinine ≥ 0.3 mg/dl (44 µmol/L) within 48 h, or relative increase of ≥ 1.5–1.9 compared to baseline, within 7 days or a diuresis < 0.5 ml/Kg of weight for at least 6 h after contrast agent administration [9].

It is important to consider other renal impairment causes or predisposing factors for PC-AKI, such as hypotension, hypovolemia and association with nephrotoxic drugs.

The pathophysiology of PC-AKI has been studied in several animal and human studies, but it is still not completely understood [10, 11].

Contrast agent effect is mediated by two main mechanisms that concurrently act: direct toxicity on renal tubular cells and ischemic damage from tissue hypoxia [10–17].

Patients at risk for PC-AKI have some risk factors (advanced age, diabetes, renal insufficiency, anemia, cardiovascular disease) that result in reduced renal vasodilatory capacity (reduced functional renal reserve), associated with endothelial dysfunction and atherosclerosis [18–20].

The incidence of PC-AKI varies depending on the modality of administration:

- Intravenous administration is not associated with an increased incidence of PC-AKI in subjects with eGFR ≥ 30 ml/min/1.73 m2 [21–26].
- In intra-arterial administration with second-pass renal exposure, the contrast agent injected into the right heart and pulmonary arteries or directly into the carotid, subclavian, brachial and mesenteric arteries, as well as into the subrenal aorta and iliac and femoral arteries, reaches the renal arteries after dilution; this administration has the same risk than intravenous administration [8];
- In intra-arterial administration with first-pass renal exposure, the contrast agent injected into the left heart, thoracic and suprarenal abdominal aorta, and selectively into the renal arteries, reaches the renal arteries during the first pass; patients undergoing these procedures have frequently comorbidities, and it is difficult to distinguish the effects due to the administration of contrast agent from those due to other concomitant causes [8, 25, 26], such as, for example, in endovascular interventional procedures, the risk of cholesterol emboli [8].

## Identification and staging of patient at risk of PC-AKI

Risk factors for PC-AKI have been mainly studied in patients undergoing cardiologic procedures (coronarography and percutaneous coronary intervention—PCI); so, those results cannot be applied to the intravenous administration [27–29]. Anyway, preexisting renal insufficiency represents the major risk factor, followed by diabetes, advanced age, heart failure, hypovolemia, myocardial infarction, anemia, peripheral vasculopathy, and the use of nephrotoxic drugs (some antibiotics, some anticancer drugs, NSAIDs, cyclosporine). In addition, the presence of pre- and intra-procedure hypotension, the use of a cardiac counterpulsator, and multiple, close (< 72 h) administrations have been identified as risk factors. Recently, hyperglycemia [30], metabolic syndrome, hyperuricemia [31], hyperhomocysteinemia [32] and atrial fibrillation [33] have also been associated with worsening renal function, while there is no evidence that the use of ACE inhibitor drugs or angiotensin receptor blockers is associated with an increased incidence of PC-AKI [34].

Diuretic drugs may be a risk factor in hypovolemia, but not when associated with adequate hydration or in the heart failure.

Myeloma and other paraproteinemias are not generally risk factors, but may lead to PC-AKI if associated with dehydration, renal failure or hypercalcemia [35, 36].

In urgency setting, patients with acute myocardial infarction may have a very high risk of PC-AKI [37]. In these cases, the risk is related to hemodynamic instability of the patient, to the lack of time for prophylaxis (see below) and to the high doses of contrast agent needed [38].

It is important to underline that serum creatinine alone is not a good index of the patient’s renal function because its value increases significantly only when the GFR is reduced to 50% and that serum creatinine represents the balance between creatinine produced by the muscles and that eliminated by the kidney.

The measure of glomerular renal filtrate (GFR) obtained from serum creatinine with the MDRD (Modification of Diet in Renal Disease Study) formula [39] or with the CKD—EPI (Chronic Kidney Disease—Epidemiology Collaboration) formula [40] represents the marker of renal function to be used for screening the renal risk of patients. ESUR (European Society of Urogenital Radiology) guidelines (GLs) recommend the use of the CKD—EPI formula [8].

The GFR measurement obtained from the two formulas is normalized to 1.73 m^2^ body surface area and does not consider the patient’s body weight.

For these reasons, it may be suggested to measure renal filtrate with the Cockroft–Gault (CG) formula [41] (which consider body weight) in patients with significantly reduced body weight, in whom the MDRD or CKD EPI formulas overestimate renal filtrate compared with the CG formula.

In pediatric patients, the Schwartz formula has been validated as more reliable than the previous formulas used [8].

It is important to define what level of GFR is associated with the risk of PC-AKI.

The 2018 ESUR LGs [8] indicate an eGFR of less than 45 ml/min/1.73 m^2^ for intra-arterial administrations with first renal pass and less than 30 ml/min for intravenous and intra-arterial administrations with second pass.

It is recommended that baseline eGFR be assessed on creatinine value performed within 7 days in patients with unstable or hospitalized renal function, whereas a 3-month interval is considered correct in other patients [8].

## Key points

**Table:** 

Serum creatinine alone is not a good index of a patient’s renal function because its value increases significantly only when the GFR is reduced to 50%
The GFR value obtained from serum creatinine with the MDRD formula or the CKD—EPI formula is the marker of renal function to use in screening patients for renal risk. The ESUR GLs recommend the use of the CKD—EPI formula. The GFR measurement obtained from the two formulas is normalized to 1.73 m^2^ of body surface area and does not consider the patient’s body weight
Renal filtrate can be measured with the Cockroft–Gault formula in patients with significantly reduced body weight
We recommend assessing baseline eGFR on creatinine value performed within 7 days in patients with unstable or hospitalized renal function, whereas a 3-month interval is considered correct in other patients
Chronic renal failure is considered the major risk factor in the oncological patient, but only for an eGFR < 30 ml/minute/1.73 m^2^ as measured by the Cockroft–Gault formula

### The optimal dose of contrast agent

It is well known that the dose of contrast agent administered is a risk factor for PC-AKI, and in fact all guidelines recommend to avoid high doses, especially in patients at risk.

The optimal dose to be proposed probably does not exist, and in the literature there are reports that even a small dose can cause PC-AKI in high-risk patients. Therefore, it is recommended to administer the minimum dose sufficient to obtain the diagnostic information [42–45].

## Renal damage prevention

### Hydration

Hydration represents the gold standard of the preventive therapy of PC-AKI [46–52].

The efficacy of hydration is explained by the protective action of circulating volume expansion against vasoconstriction due to contrast agent and by increased diuresis, which reduces the direct contact toxicity of the drug on renal tubular cells [50, 51].

In recent years, there has been interest in the literature in the use of sodium bicarbonate (NaBic), with discordant results [53–61].

In accordance with ESUR guidelines [8], intravenous (e.v.) hydration is recommended as a preventive measure for patients with moderate risk of PC-AKI (e.v. or intra-arterial administration with second renal passage with GFR < 30) using either NaBic 1.4% 3 ml/Kg/hr for 1 h before administration, or saline 1 ml/Kg/hr for 3–4 h before and 4–6 h after administration.

In patients with intra-arterial administration with renal first pass and GFR < 45 ml/min/1.73 m^2^, it is recommended intravenous hydration with NaBic 1.4% 3 ml/kg in the hour before administration, maintained at 1 ml/kg/hr for 4–6 h thereafter, or saline 1 ml/Kg for 3–4 h before and 4–6 h thereafter [8].

The meta-analysis by Meier [59] and the more recent one by Zhang [60] show that NaBic is more useful than saline when there is no time to perform prolonged hydration, and that could be more indicated in emergency procedures.

For outpatients, the need for intravenous hydration may represent a logistical problem for both departments of radiology and nephrology.

For this reason, it is recommended that each hospital has a protocol for the prevention of PC-AKI for both outpatients and inpatients at risk that also considers the logistical aspect (where to perform the prevention intravenous therapy).

### Medical therapies in the prevention of PC-AKI

#### *N*-Acetylcysteine and statins

To date, there is no evidence on the efficacy of NAC toward PC-AKI prevention; the 2018 ESUR guidelines do not recommend its use [8].

Studies on patients undergoing cardiological procedures have suggested that the use of statins at high dosage (atorvastatin 80 mg or rosuvastatin 20–40 mg) could reduce the risk of PC-AKI [8, 62, 63]. However, the relevant bias of these observations led ESUR guidelines to advice against their use [8].

### Radiologic procedures performed in non-deferrable emergencies

Because of the efficacy of intravenous hydration with 1.4% NaBic performed 1 hour before iodine-based contrast agent infusion, this strategy can be proposed in radiological examinations/procedures performed in urgency in patients at risk of PC-AKI [59, 60].

In patients at increased risk of PC-AKI, the diuresis assessment and monitoring should be considered. It is recommended in hospitalized patients at increased risk, because of the presence of comorbidities added to preexisting renal insufficiency, such as heart failure, neoplasms and surgical issues, to achieve a diuresis ≥ 100 mL/hour before administration of the contrast agent and in the following 12 h.

In patients with heart failure, hydration should be combined with the diuretic to achieve an adequate increase in diuresis, thus avoiding worsening the edema status.

### Patients with a very high risk of PC-AKI

An important and topical issue, considering the higher morbidity and age of patients who must undergo interventional examinations and procedures with contrast agent, concerns the mode of prevention in patients with severe preexisting renal insufficiency (GFR < 20 ml/min/1.73 m^2^) in whom the use of contrast agent represents a risk of further worsening and possible entry into dialysis.

In patients with severe heart failure (NYHA—New York Heart Association class 3–4) or with preexisting severe chronic renal failure (GFR ≤ 15, CKD 5), the hydration protocol should be “personalized” and decided on a multidisciplinary setting [8].

There is no evidence of the effectiveness of dialysis methods toward the prevention of PC-AKI [64]; for this reason, it is not recommended [8].

In these patients, especially if subjected to procedures with intra-arterial administration with first renal passage, the problem is unsolved and the issue is still open; for this reason, it is recommendable that each center should have an individualized PC-AKI prevention protocol on this type of patients.

## Key points

**Table:** 

Hydration/volume expansion represents the gold standard of PC-AKI preventive therapy, keeping in mind that any intervention must be proportionate to the patient’s overall risk;
Intravenous hydration is recommended as a preventive measure for patients with moderate risk of PC-AKI (intravenous or intra-arterial administration with second renal passage with GFR < 30) using either Na bicarbonate (NaBic) 1.4% 3 ml/Kg/hr for 1 h prior to administration, or saline 1 ml/Kg/hr for 3–4 h before and 4–6 h after administration;
In patients with intra-arterial administration with renal first pass and GFR < 45 ml/min/1.73 m^2^, we recommend intravenous hydration with NaBic 1.4% 3 ml/kg in the hour before the administration, maintained at 1 ml/kg/hr for 4–6 h thereafter, or with physiologic 1 ml/Kg for 3–4 h prior and for the next 4–6 h;
Two meta-analyses by Meier show that NaBic is more useful than saline when there is no time to perform prolonged hydration and thus may be more appropriate in emergency procedures
The prevention of PC-AKI in patients at very high risk (due to comorbidity and to the procedure itself) undergoing procedures with intra-arterial administration with first renal passage remains debated, and for this reason we recommend that each center should have an individualized prevention protocol for this type of patients

### Oncological patient

The development of PC-AKI has often a multifactorial genesis because of the association of other risk factors for renal damage:

1. Advanced age;
2. Comorbidities;
3. Toxicities potentially impacting renal function (such as dehydration due to diarrhea, stomatitis, vomiting);
4. Preexisting chronic renal failure [65];
5. Use of potentially nephrotoxic agents, such as cytotoxic chemotherapeutics, some molecularly targeted drugs, immunotherapy, NSAIDS and bisphosphonates.

It is essential to consider for each patient the risk–benefit ratio of contrast agent administration, even in those particularly at risk of developing PC-AKI, discussing it in multidisciplinary meetings.

Hydration is recommended to prevent PC-AKI from contrast agent infusion even in oncological patients at risk.

Finally, regarding the management of cancer therapy in patients who have to perform radiological examinations with contrast agent (especially if already suffering from chronic renal failure), the behavior to be kept depends on the type of therapy in progress:

1. Patients treated with cytotoxic chemotherapy or with potentially nephrotoxic chemotherapy (especially if containing cisplatin) who receive iodine-based contrast agent are at higher risk of developing PC-AKI [65, 66]. It is recommended that 5 to 7 days elapse between administration of either. No other data are available in the literature regarding other chemotherapeutics, so we recommend not discontinuing them before a CT with contrast agent.
2. Patients treated with molecularly targeted drugs and immunotherapy: It is not recommended to discontinue treatment at CT scans with contrast agent.
3. Patients treated with bisphosphonates: The nephrotoxicity of bisphosphonates is now well known in the oncology setting. We recommend a 14-day interval between zoledronic acid and iodine-based agent administration.

## Key points

**Table:** 

Hydration is the only recommended measure to prevent PC-AKI from contrast agent infusion even in the high-risk oncological patient;
Oncological patients treated with potentially nephrotoxic chemotherapy (especially if containing cisplatin) who receive iodine-based contrast agent are at high risk of developing PC-AKI. It is recommended that 5 to 7 days elapse between the administration of cisplatin and the contrast agent. There are no data in the literature regarding other chemotherapeutics, which are therefore not recommended to be discontinued before a CT with contrast agent;
In patients treated with molecularly targeted drugs and immunotherapy, renal damage due to these drugs has a significantly lower incidence than with chemotherapy; it is not recommended to discontinue treatment at CT scans with contrast agent
In patients treated with bisphosphonates, it is recommended that an adequate interval be maintained between their administration and that of the contrast agent. A 14-day interval between zoledronic acid and iodine-based agent administration is recommended

### Gadolinium nephrotoxicity risk

The risk of PC-AKI after an MRI examination with gadolinium (Gd) is very low when the contrast agent is used in the approved doses (0.1 mmol/Kg). The data available in the literature allow to minimize the potential nephrotoxicity, but not to exclude it at all, and instead put the focus on renal function because when it is reduced, it may “facilitate” the intrinsic toxicity of gadolinium in inducing systemic nephrogenic fibrosis (NSF) or gadolinium deposition disease (GDD) [67–70].

At present, it can be reasonably supposed that the use of gadolinium chelates cannot be considered a risk factor for PC-AKI in patients with eGFR ≥ 30 ml/min/1.73 m^2^ [46].

Gd-based MDCs (GdCAs) have similar pharmacokinetics to iodine-based contrast agents.

### Risk of nephrogenic systemic fibrosis

Nephrogenic systemic fibrosis (NSF) was first described in 2000 as a specific and rare skin disorder in 15 patients who had renal insufficiency, hence the name nephrogenic fibrosis [71].

Renal insufficiency represents only one of the risk factors related to NSF, since neither the presence of renal insufficiency nor a threshold value of GFR reduction is recognized as risk classification criteria.

Gadolinium deposition in tissues following exposure to this agent has been documented and associated with factors such as agent stability, high and repeated doses. Impaired renal function of the patient is a predisposing factor because it slows the excretion of the molecule and promotes its deposition.

All these observations represent the background of the current guidelines that recommend to know the value of glomerular filtrate and the stability of renal function before the administration of Gd, suggesting greater caution when the eGFR < 30 ml/min and the indication for the execution of the hemodialysis session immediately after the administration of GdCA in patients already under chronic dialysis treatment, since there is no evidence that the hemodialysis session absolutely prevents the deposition of tissue gadolinium and therefore reduces the risk of complications [72–78].

## Dialysis after contrast agent in patients undergoing chronic dialysis treatment

It is not recommended to coordinate dialysis after iodine-based contrast agent infusion or to provide an additional dialysis session to remove it [8].

In contrast to iodine-based agent, as mentioned previously, in agreement with the same ESUR guidelines, it is suggested that MRI examination with Gd be scheduled concurrently with hemodialysis treatment and that contrast agent be removed as quickly as possible with any additional dialysis session [79].

## Abbreviations

CI-AKI: Contrast-induced acute kidney injury
CKD-EPI: Chronic kidney disease—epidemiology collaboration
CG: Cockroft–Gault formula
eGFR: Estimated glomerular filtration rate
ESUR: European Society of Urogenital Radiology
KDIGO: Kidney Disease: Improving Global Outcomes
MDRD: Modification of diet in renal disease study
NaBic: Sodium bicarbonate
NSF: Nephrogenic systemic fibrosis
NSAID: Nonsteroidal anti-inflammatory drug
NYHA: New York heart association
PC-AKI: Post-contrast acute kidney injury
PCI: Percutaneous coronary intervention
